# Laparoscopic Curative Resection for Right-Sided Colonic Tumors: Initial Experience From a Specialized Cancer Hospital of a Developing Country

**DOI:** 10.7759/cureus.9465

**Published:** 2020-07-29

**Authors:** Awais Naeem, Osama Shakeel, Ijaz Ashraf, Sheryar Riaz, Ihtisham Haq, Muhammad F Shah, Abdul Wahid Anwer, Irfan ul Islam Nasir, Awais Amjad, Shahid Khattak, Aamir Ali Syed

**Affiliations:** 1 Surgical Oncology, Shaukat Khanum Memorial Cancer Hospital and Research Centre, Lahore, PAK

**Keywords:** laparoscopic colon resection, laparoscopic right hemicolectomy, extended right hemicolectomy, complete mesocolic excision, central venous ligation, laparoscopic resection of right-sided colonic carcinoma

## Abstract

Introduction

Laparoscopic colonic resection is increasingly becoming popular worldwide and aims to provide curative resection in addition to the inherent benefits of laparoscopic surgery. The aim of this study was to evaluate the long-term outcomes of laparoscopic right hemicolectomy in a Pakistani cohort of patients.

Methods and procedures

We retrospectively analyzed the medical records of all patients who presented to our hospital with the diagnosis of right-sided colon carcinoma from January 2010 to December 2018 and underwent laparoscopic right or extended right hemicolectomy. Demographics, operative findings, histopathology report, and follow-up of patients were recorded and the analysis was performed on Statistical Packages for the Social Sciences (SPSS) Version 20 (IBM Corp, Armonk, NY).

Results

Seventy-five patients were included, 56 (74.7%) of whom were males and 19 (25.3%) were females. The median age was 52 years (range 25-82 years). The median hospital stay was five days (Range 3-13 days). The median blood loss was 70 milliliters and the mean operative time was 195.5±77.6 minutes. Laparoscopic extended right hemicolectomy was performed in 23 (16.67%) patients and standard right hemicolectomy in 52 (83.33%) patients. Most (72%) of the patients had a pathological T3 tumor, and the majority (61.3%) of the patients had no nodal involvement (pN0). The mean number of lymph nodes removed was 20+8. The median numbers of involved lymph nodes were 1.14+2.19. All the patients had R0 resection. Postoperatively, two patients had pelvic collection, and there was no 30-day mortality. Local recurrence occurred in four patients and distant metastases were observed in nine patients. The median follow-up in our study was 40.5±18.35 months. The median disease-free survival was 42±2.17 months and the median overall survival was 44±2.16 months.

Conclusion

Our experience with laparoscopic right colon resections has confirmed the safety and feasibility of the procedure.

## Introduction

Colorectal cancer (CRC) is the third most deadly and fourth most commonly diagnosed cancer in the world [1]. Colonic resection is the only curative option available for patients with colon cancer. Laparoscopic colonic resections were first described by Jacob in 1991 [2]. The Clinical Outcomes of Surgical Therapy Study Group (COST) [3] and Colon Cancer Laparoscopic or Open Resection (COLOR) [4] trials have shown longer operating times but quicker recovery for laparoscopic resections and no difference in morbidity, mortality, recurrence, or survival. The Medical Research Council Conventional versus Laparoscopic Assisted Surgery in Colorectal Cancer (MRC CLASICC) trial determined equivalent perioperative and oncological outcomes after colorectal resections [5-6].

The concept of total mesorectal excision (TME) introduced by Heald has transformed the oncologic outcomes of rectal cancer surgery [7-9]. The basic principle of TME is dissection in the ‘Holy plane’ between the embryologic mesorectal and parietal fascia. This en bloc resection specimen contains the draining lymphatics, lymph nodes, and blood vessels through which the tumor may spread, hence minimizing local recurrence rates and improving survival.

In 2009, Hohenberger described the concept of complete mesocolic excision (CME) for colonic cancer [10]. The concept of CME encompasses dissection in the embryological plane, central vascular tie to remove all lymph nodes along the vessels, and adequate proximal and distal margins of the bowel [10]. Several studies have advocated the efficacy of CME in terms of improved oncological outcomes [11-12].

As per our knowledge, only a few centers in Pakistan are performing laparoscopic right hemicolectomies owing to the dearth of resources and expertise, and no experience of laparoscopic right hemicolectomy has yet been published from Pakistan. Therefore, we are reliant on Western literature and do not know the long-term outcomes of laparoscopic right-sided colon cancer resections in Pakistan. Shaukat Khanum Memorial Cancer Hospital and Research Centre (SKMCH&RC) is a specialized cancer hospital that receives the largest number of colorectal cancer patients in the country.

The aim of this study is to provide an analysis of the patient characteristics and long-term outcomes after laparoscopic right hemicolectomy in a Pakistani cohort of patients. This will provide a reference point for physicians to counsel patients about the outcomes in Pakistan and help define areas of potential improvement in the future.

## Materials and methods

All patients referred to our hospital has a detailed history and physical examination in the walk-in-clinic. All the patients undergo colonoscopy and biopsy, baseline carcinoembryonic antigen (CEA) levels, computed tomography (CT) chest abdomen, and pelvis and multidisciplinary team (MDT) discussion.

All patients undergoing laparoscopic right and extended right hemicolectomies between January 1, 2010, and December 31, 2018, at Shaukat Khanum Memorial Cancer Hospital and Research Center (SKMCH&RC) were selected. It is a retrospective study with convenience sampling. Those patients who underwent open right hemicolectomy or had a conversion from laparoscopy to open during the procedure were excluded. Ethical approval was sought from the Institutional Review Board (IRB) of SKMCH&RC. The study complies with the SKMCH&RC guidelines on research involving human subjects.

We are a paperless hospital and all patient data is put in real-time into a computerized hospital information system (HIS). The hospital has a unique computerized patient management system developed in-house that collects all patient information in real-time, including patient demographics, investigations, MDT discussions, nursing assessments, outpatient, operative notes, and postoperative outcomes. As the data is collected in real-time and stored, it allows for an accurate retrospective review of the data. Variables recorded were age, gender, pre-operative histopathology, clinical staging, treatment received, type of surgery, pathological stage, surgical outcomes of the procedure, and oncological outcomes. Calculations were performed with the Statistical Package for the Social Sciences (SPSS 20) for Windows Version 20 (IBM Corp, Armonk, NY) statistical software.

At our institution, laparoscopic right hemicolectomy is performed by two surgeons using three or four working ports in addition to the supra-umbilical camera port. The ‘medial to lateral’ approach is used, as the mesocolon is incised along the mesenteric axis. The ileocolic vessels are clipped and transected close to the superior mesenteric vein. After exposing the mesocolic interface, wide separation is achieved between the pancreatic head and the transverse colon. Dissection then proceeds along the superior mesenteric vein, exposing the gastrocolic trunk of Henle. The middle colic artery is then identified as it rises from the superior mesenteric artery and is transected at the root of its right branch in case of a standard right hemicolectomy or at the origin of the middle colic artery in case of an extended right hemicolectomy. This is accompanied by lymph node dissection, taking care to preserve the left branch of the middle colic artery. The fascia is detached between the omentum and transverse mesocolon and the hepatic flexure is mobilized. Mobilization of the bowel is completed. The specimen is extracted by a mini-laparotomy (small midline or right upper quadrant transverse incision), and an extracorporeal, stapled, side-to-side anastomosis is performed.

## Results

A total of 75 patients were operated laparoscopically, of which 56 (74.7%) were males and 19 (25.3%) were females. The median CEA level was 4.1 ng/ml; abdominal pain (41.3%) and weight loss (36%) were the most common symptoms; and 53.3% of the patients were anemic (Table 1).

**Table 1:** Demographic variables ASA: American Society of Anesthesiologists; IHD: Ischaemic heart disease

VARIABLES		VALUES
Age – Median		52 +/- 12.63
Age – Category	Below 40 years	17 (22.7%)
	Above 40 years	58 (77.3%)
Gender	Male	56 (74.7%)
	Female	19 (25.3%)
ASA grade	ASA I	9 (12.0%)
	ASA II	55 (73.3%)
	ASA III	11 (14.6%)
Family History	Yes	19 (25.3%)
	No	56 (74.7%)
Comorbidities	Hypertension	12 (16.0%)
	Diabetes	9 (12.0%)
	IHD	1 (1.3%)
Addiction	Smoking	30 (40.0%)
	Tobacco chewing	14 (18.6%)
	Alcoholism	6 (8.0%)
Symptoms/Signs	Anemia	40 (53.3%)
	Abdominal pain	31 (41.3%)
	Weight loss	27 (36.0%)
	Altered bowel habits	11 (14.6%)
	Melena	5 (6.66%)
Demographic distribution	Punjab	49 (65.3%)
	Khyber Pakhtunkhwa	18 (24%)
	Afghanistan	5 (6.63%)
	Sindh	3 (4%)

Standard right hemicolectomy was performed in 69.3% (n=52) of the patients while extended right hemicolectomy was performed in 30.7% (n=23) of the patients. The median blood loss during surgery was 50±70.32 milliliters (ml) and the median operative time was 195.5±77.6 minutes. Seventy-two patients underwent elective surgeries. The median hospital stay was five days (Range 3-13 days).

The caecum (29.3%, n=22) was the commonest site of the tumor. Pathological T3 (72%, n=54)) stage was the commonest T stage. Most (48%, n=36) of the patients had no nodal involvement. The mean total number of lymph nodes retrieved was 20 + 8. The median numbers of involved lymph nodes were 1.14+2.19. Tumor characteristics and surgical outcomes are shown in Table 2. All the patients had R0 resection. The specimen length retrieved was a median length of 30+10.0 cm. The median length of the proximal and distal margins was 12±8.6 cm and 10±6.89 cm. Lymphovascular invasion was seen in two patients (Table 2).

**Table 2:** Tumor characteristics and surgical outcomes Tumor: T; Nodal: N

Stage of disease	Stage I	1 (1.3%)
	Stage II	6 (8.0%)
	Stage III	54 (72.0%)
	Stage IV	14 (18.6%)
Carcinoembryonic antigen	Less than 5 ng/ml	42 (56.0%)
	More than 5 ng/ml	33 (44.0%)
Mode of surgery	Elective	72 (96.0%)
	Emergency	3 (4.0%)
Site of tumor	Cecum	22 (29.3%)
	Ascending colon	19 (25.3%)
	Hepatic flexure	16 (21.3%)
	Transverse colon	15 (20.0%)
	Appendix	3 (4.0%)
Median duration of surgery		195.5±77.6 minutes
Median blood loss		50±70.32 ml
Median hospital stay		5 days (Range 3-13 days)
Pathological T stage	T2	6 (8.0%)
	T3	54 (72.0%)
	T4	15 (20.0%)
Pathological N stage	N0	36 (48%)
	N1	29 (38.6%)
	N2	10 (13.3%)

There was no 30-day mortality. Six patients developed postoperative complications. Four patients developed superficial surgical site infections (SSI). Two patients developed intra-abdominal collection, for which the radiologic intra-abdominal drainage was performed.

**Figure 1:**
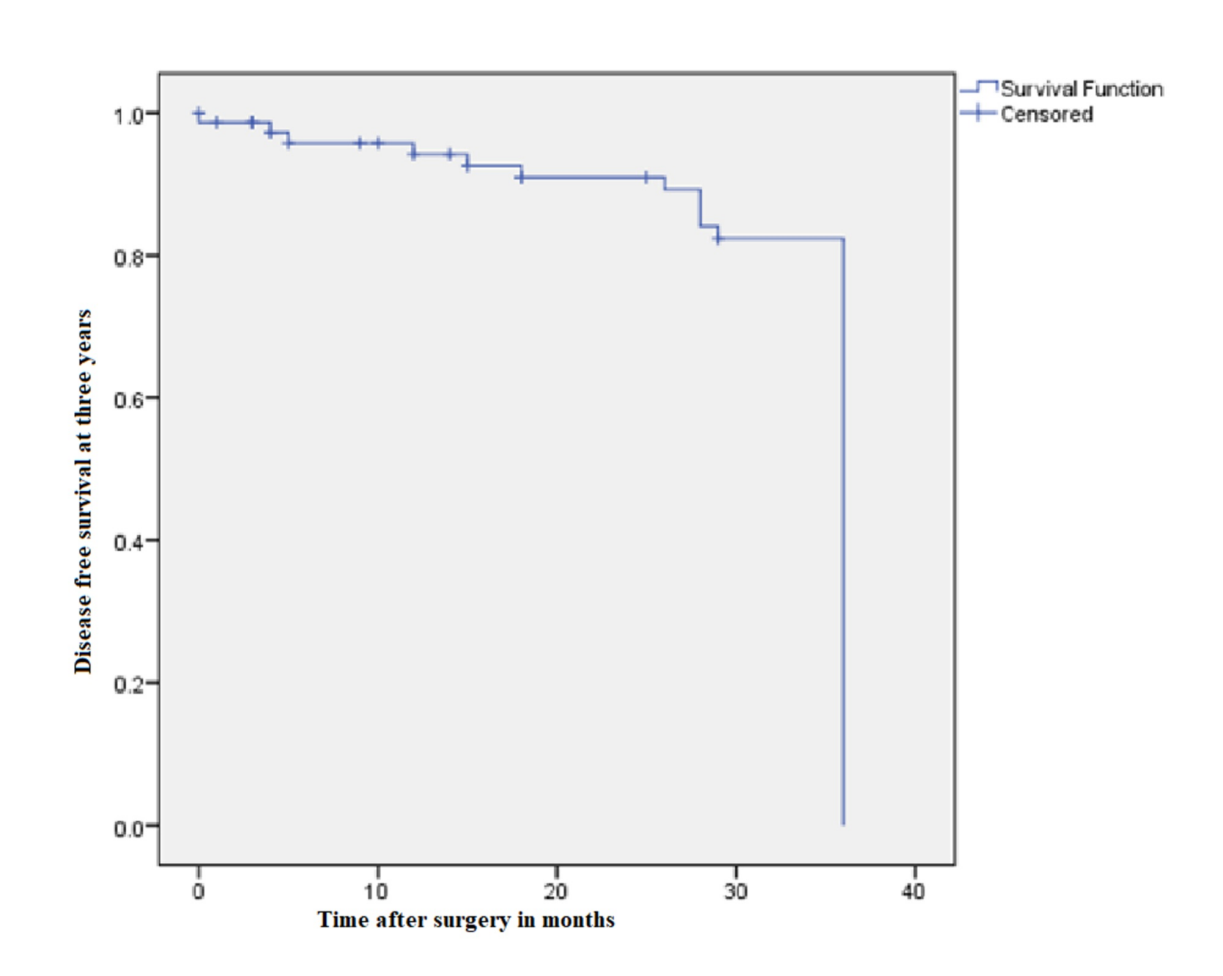
Kaplan-Meier curve showing disease-free survival at three years

**Figure 2:**
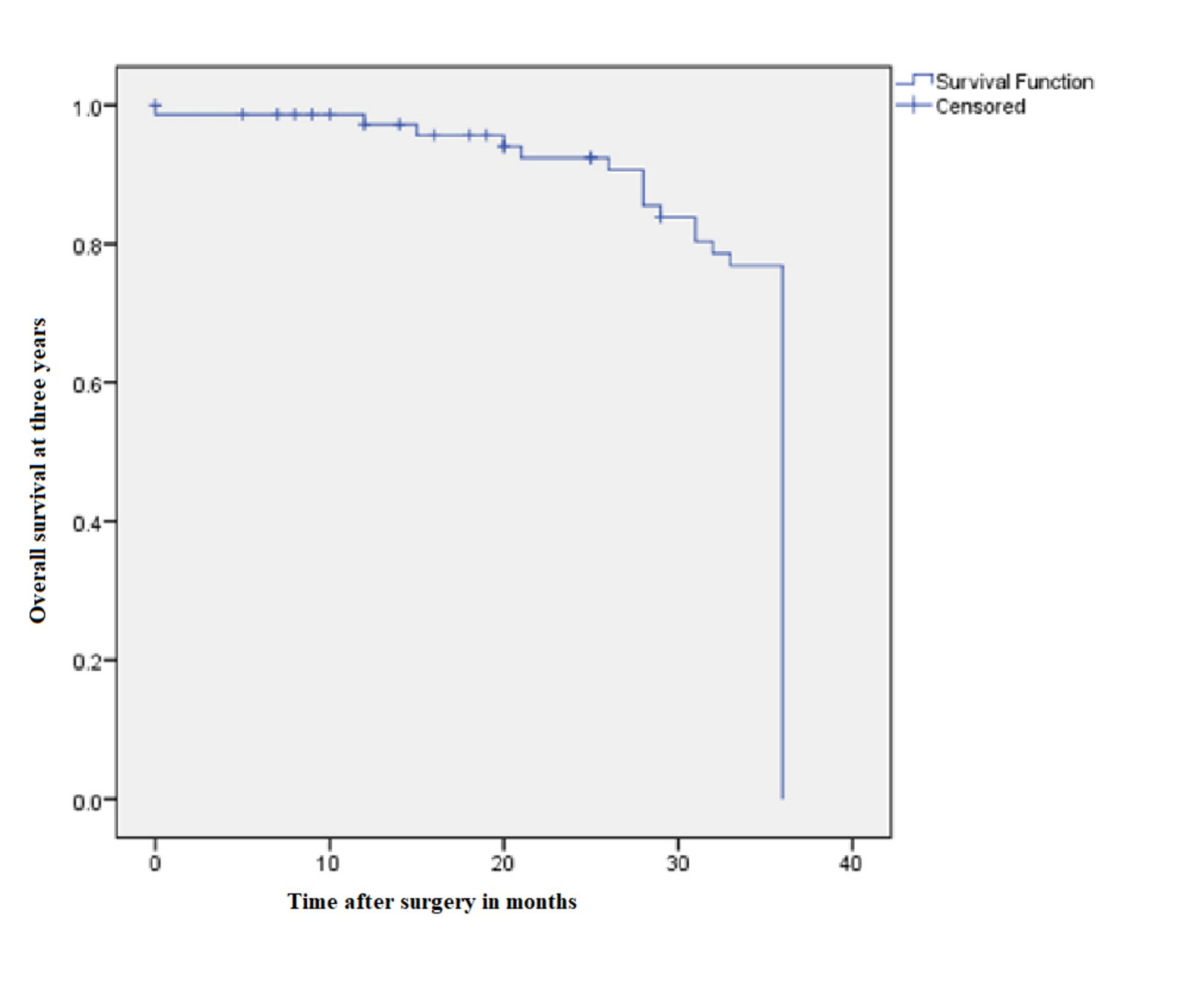
Kaplan-Meier curve showing overall survival at three years

Loco-regional recurrence was seen in four patient and they were given chemotherapy. Nine patients developed metastases. The sites of metastases were lung (3 patients), liver (4 patients), bone (1 patient), and brain (1 patient). The median disease-free survival was 42±2.17 months and the median overall survival was 44±2.16 months. The median follow-up in our study was 40.5±18.35 months (Figures 1-2).

## Discussion

The goal of curative colon cancer surgery is to remove the tumor with adequate bowel margins along with its accompanying blood supply, lymphatics, and lymph nodes. Open resections were performed until 1991 when Jacob presented his data on laparoscopic colonic resections [2]. Multiple trials have established the benefits of laparoscopy and its oncologic equivalence [3-6]. Dissection in embryological planes for rectal cancer, the so-called ‘Holy plane’ described by Heald, led to an increased disease-free and overall survival for rectal cancer patients [7-9]. The concept behind total mesorectal excision (TME) for rectal cancers is that with precise dissection, mesorectal fascia can be preserved and removed en masse with the rectum. Central venous ligation (CVL) is performed, making sure to remove the draining lymph nodes, and an adequate bowel length is also removed to remove the pericolic lymph nodes.

The concept of complete mesocolic excision (CME) was coined by Hohenberger and encompassed three components: dissection in the embryologic plane, central venous ligation (CVL), and resection of adequate bowel length [10]. For complete mesocolic excision (CME) for right-sided colon cancers, the mobilization of mesocolon is more radical as compared with the ‘standard’ right hemicolectomy. The CVL for right hemicolectomy means the exposure of superior mesenteric vessels and the anterior surface of the pancreas and ligation of the ileocolic vessel at the origin. In ‘standard’ right hemicolectomy, ileocolic vessels are ligated as per the surgeon’s convenience. At our institution, dissection is performed in the embryologic plane and adequate length of the bowel is removed. Ileocolic vessels are ligated to the right of the superior mesenteric vein.

Adequate nodal staging is crucial in the management of colorectal cancer. The patient’s prognosis is influenced by the number of retrieved lymph nodes [13-14]. D3 lymphadenectomy is being followed by many colorectal units performing colonic surgery in Asia, particularly in Korea and Japan. This technique is almost similar to CME [15-16]. The accuracy of staging and survival is greatly improved due to the higher nodal yield of CME [17-18]. West et al. reported a greater average nodal yield of 31.3 nodes for CME, as compared to that of 20 nodes for conventional right hemicolectomy [19]. Hohenberger's study also demonstrated improved survival in node-negative patients when >28 lymph nodes were removed [10]. Li-Ying et al. published that they harvested 17 lymph nodes on average [20]. The mean total number of retrieved lymph nodes in our study was 20±8. The possible explanation for this is the fact that the ileocolic vessels were ligated to the right of superior mesenteric vein thus missing the apical lymph nodes.

Recent studies comparing standard laparoscopic to open right hemicolectomy have reported a length of stay of six to 13 days [21-22]. In our study, the median hospital stay was five days (Range 3-13 days). The mean operative time in our study was 195.5±77.6 minutes, which is comparable with that of other studies [21-23]. There was no anastomotic leak or 30-days mortality after surgery in our patients. According to the studies, postoperative morbidity after CME ranges from 5.9% to 19.7% [24-25]. Four of our patients developed superficial surgical site infection and two patients developed deep organ space infection that was drained radiologically. Therefore, our postoperative morbidity rate (8%) is comparable to other studies.

Hohenberger showed the oncologic advantages of CME. With proper CME, the recurrence rate dropped to 3.6% and five-year cancer-related survival improved to 89.1% [10]. An Australian study conducted by Bokey et al. also demonstrated improved five-year overall survival from 48% to 63% and disease-specific survival from 66% to 76% after the introduction of CME/CVL [26]. Four (5.3%) of our patients developed local recurrence. Nine patients developed metastases. The sites of metastases were lung (three patients), liver (four patients), bone (one patient), and brain (one patient). The median disease-free survival was 42±2.17 months and the median overall survival was 44±2.16 months. The median follow-up in our study was 40.5±18.35 months.

The limitations of this study are its retrospective nature and small sample size. But this is the first such experience of laparoscopic right hemicolectomy being published from Pakistan and will, hopefully, pave the way for future research into the benefits of laparoscopic colonic resection.

## Conclusions

Our results showed that a structured approach to laparoscopic right-sided colonic resection is associated with a reduction in hospital stay, reasonable operative times, and acceptable morbidity. Although laparoscopic surgery is expensive and requires laparoscopic training for surgeons, laparoscopic resection is an acceptable alternative to open surgery. Laparoscopic right-sided colonic resection is a safe and effective procedure and that even in a resource-limited setup, it is possible to achieve results that are comparable to international studies. Further studies are needed to establish the long-term oncologic safety of the procedure.
